# BERT based natural language processing for triage of adverse drug reaction reports shows close to human-level performance

**DOI:** 10.1371/journal.pdig.0000409

**Published:** 2023-12-06

**Authors:** Erik Bergman, Luise Dürlich, Veronica Arthurson, Anders Sundström, Maria Larsson, Shamima Bhuiyan, Andreas Jakobsson, Gabriel Westman

**Affiliations:** 1 Swedish Medical Products Agency, Uppsala, Sweden; 2 Department of Computer Science, RISE Research Institutes of Sweden, Kista, Sweden; 3 Department of Linguistics and Philology, Uppsala University, Uppsala, Sweden; 4 Centre for Mathematical Sciences, Lund University, Lund, Sweden; 5 Department of Medical Sciences, Uppsala University, Uppsala, Sweden; Mayo Clinic Scottsdale, UNITED STATES

## Abstract

Post-marketing reports of suspected adverse drug reactions are important for establishing the safety profile of a medicinal product. However, a high influx of reports poses a challenge for regulatory authorities as a delay in identification of previously unknown adverse drug reactions can potentially be harmful to patients. In this study, we use natural language processing (NLP) to predict whether a report is of serious nature based solely on the free-text fields and adverse event terms in the report, potentially allowing reports mislabelled at time of reporting to be detected and prioritized for assessment. We consider four different NLP models at various levels of complexity, bootstrap their train-validation data split to eliminate random effects in the performance estimates and conduct prospective testing to avoid the risk of data leakage. Using a Swedish BERT based language model, continued language pre-training and final classification training, we achieve close to human-level performance in this task. Model architectures based on less complex technical foundation such as bag-of-words approaches and LSTM neural networks trained with random initiation of weights appear to perform less well, likely due to the lack of robustness that a base of general language training provides.

## Author summary

Reports of suspected adverse drug reactions are important for drug safety. However, a high influx of reports poses a challenge for regulatory authorities as a delay in identification of previously unknown adverse drug reactions can potentially be harmful to patients. In this study, we show that such reports can be automatically classified into serious and non-serious cases through the use of natural language processing. We have compared four different system architectures for this task, and show that an approach based on a pre-trained Swedish language model (BERT) shows close to human-level performance. Prospective testing suggests that the model performance estimates are relevant for real-world deployment.

## Introduction

At time of authorisation, the safety profiles for new medicinal products are limited to the adverse drug reactions (ADRs) frequent enough to be captured within the clinical development programme, while knowledge of more rare side effects can be limited by the size and inclusion criteria of the pivotal trials. Hence, systematic collection of safety data post authorisation is of great importance to further develop the safety profile.

In Sweden, reports of suspected ADRs are received by the Swedish Medical Products Agency (MPA) in electronic form or via paper forms. Reporters can be both healthcare professionals and patients/consumers. Along with a description of the suspected reaction, the reports also include data on whether the reaction led to death, was life threatening or caused a congenital malformation, hospitalization or prolonged hospitalization, permanent disability or damage, or other important medical events, in line with the definition of a serious ADR [1].

All incoming reports of suspected ADRs are triaged and processed in order of priority by assessors at the MPA, which allows the serious reports to be assessed with priority. One challenge is that a report describing a potentially serious event will not be prioritised for assessment, if it is not labelled as such at the time of reporting.

In this study, we propose to use natural language processing (NLP) to predict whether a report is of serious nature based solely on the free-text fields and adverse event terms in the report, potentially allowing reports mislabelled at time of reporting to be detected and prioritized for assessment. We consider four different Swedish NLP models at various levels of complexity and bootstrap their train-validation data split to eliminate random effects in the performance estimates and conduct prospective testing to avoid the risk of data leakage.

Recently, NLP has undergone a paradigm shift from developing dedicated feature sets and designing very specific architectures to accomplish different tasks such as sentiment analysis, syntactic parsing or part-of-speech tagging to exploiting large pre-trained models such as BERT [2] for their general language capabilities and fine-tuning them for many different tasks. In the wake of even larger generative models like GPT-3, this pre-training and fine-tuning setup is now being replaced by prompting requiring none or very few examples to obtain promising performance [3]. However, results on ScandEval, a recently published benchmark of NLP tasks in several Scandinavian languages [4], show that multiple Swedish and Norwegian BERT models on average perform better in Swedish tasks than generative models including GPT-4 [5] and that large Swedish BERT models along with GPT-4 and GPT-3.5 turbo are among the top for classification tasks in Swedish such as sentiment analysis and linguistic acceptability [6].

In addition, large generative models are typically quite demanding in terms of memory and computational requirements, and we cannot process our reports on outside servers due to privacy concerns, which is why we consider a BERT model as the largest architecture for our experiments.

Specifically, we consider two transformer-based architectures using a Swedish BERT language model, one bag-of-words approach, and one based on LSTM modelling.

## Related work

While the idea to automatically classify incoming reports is not new, previous work has considered slightly different definitions of the classification outcome–importance or seriousness–to meet different needs:

Muños *et al*. [7] predict pharmacovigilance utility of individual case safety reports (ICSRs), i.e., whether a report is likely to be included in a pharmacovigilance review, based on a range of report meta-data and some language-based features such as the length of the narrative and the presence of a set of curated narrative terms.

Lieber *et al*. [8] train a bagging classifier of decision trees for the triage of Dutch ICSRs that require thorough clinical review. Their final model uses a set of 175 features including general information about patient and case, such as age, gender, weight, drug names, and seriousness information on the case as well as binary features on word occurrence for a selection of words deemed relevant by pharmacovigilance experts in the free-text fields and the length of text fields.

In contrast to both these approaches, our approach predicts the seriousness of the report using only textual features and is agnostic of the medicinal product beyond any information present in the free-text field.

Most closely related to our objective, Routray et al. [9] use LSTMs initialized on pre-trained GloVe embeddings on the PubMed corpus to automate binary seriousness classification, assigning specific seriousness categories and identifying terms in the reports to support the seriousness category using only the free-text narrative, the reported AEs and MedDRA preferred terms.

Létinier et. al. [10] propose a pipeline for automatic identification and seriousness classification of ADRs in French free text reports from a single French pharmacovigilance centre. They test a range of different machine learning models, both more conventional models such as logistic regression, support vector machines and random forests as well as deep learning models and obtain promising results for ADR identification using gradient boosting trees, whereas the performance of all models was much lower for seriousness classification, which is likely due to the very limited amount of training data. The amount of training data is also cited as a possible reason the deep models performed worse than boosting and–along with the lack of a French BERT model pretrained on biomedical text–as one of the reasons against trying a French BERT model.

On a larger corpus of French annotated ICSRs, Martin et. al. [11] present a continuation of the work by Létinier et. al. 2021, this time comparing gradient boosting trees and general-domain transformer models–XLM [12] for ADR identification and gradient boosting trees using CamemBERT [13] embeddings for seriousness classification. The gradient boosting models use TF-IDF word vectors and structured features as input for the first task and FastText word embeddings trained on French medical text for the second. They observe the models to perform near identical on both tasks and on internal as well as external evaluation data and observe an improvement in both tasks compared to their previous work (albeit on different evaluation data) and argue that both approaches are balanced in terms of their strengths and weaknesses, because the gradient boosting trees obtain additional structured features in the identification task and have domain-specific embeddings in the classification task, whereas the transformer models are more powerful, but not adapted to the biomedical domain. The less computationally demanding models using boosting and TF-IDF and FastText are in use by the French national health authorities.

Applications in domains with language that differs strongly from the language represented in general pre-trained language model tend to benefit from language models adapted to that domain.

For the English language, a range of such domain-specific or mixed-domain models exists for biomedical and clinical language. These include PubMedBERT [14], a BERT model pre-trained from scratch on PubMed abstracts, BioBERT [15], initialized from the original BERT-base model [2] and further pre-trained on PubMed abstracts and PubMed Central full-text articles, BlueBERT [16] also initialized from BERT-base and then pre-trained on PubMed abstracts and de-identified clinical text, ClinicalBERT [17], based on BioBERT and further pre-trained on clinical text, SciBERT [18], trained from scratch on biomedical publications and computer science publications, and PharmBERT [19] specifically trained on drug labels.

These models have been shown to outperform general-domain pre-trained language models such as the original BERT base model [2] on in-domain NLP tasks such as named-entity recognition, relation extraction, question answering and document classification [14].

For the purposes of our study, however, we did not have access to an existing domain-specific model for Swedish biomedical text. Instead, we chose a BERT model that had at least part of its pretraining data consist of medicinal product information in Swedish.

## Methodology

### Data

**Fig 1:**
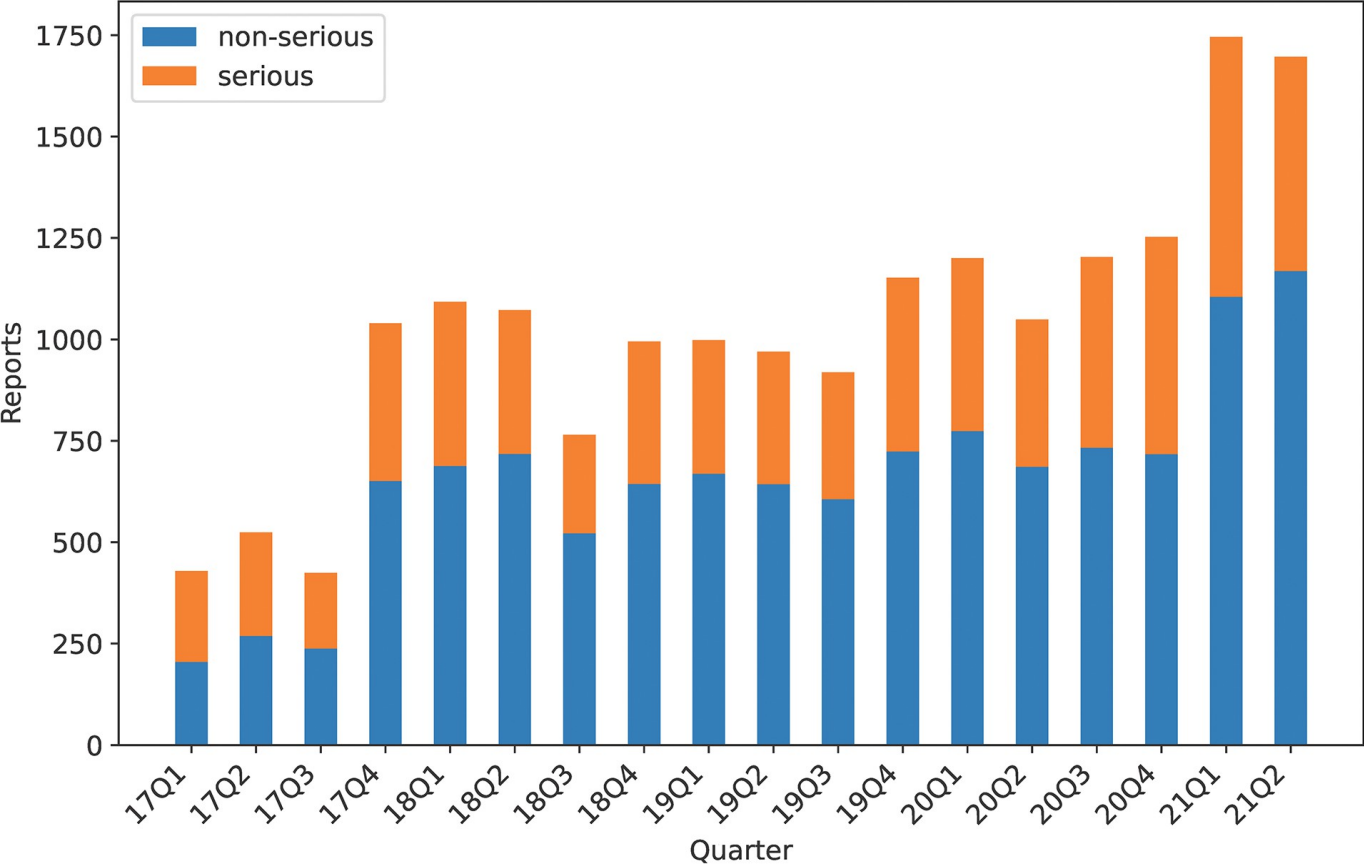
Current dataset quarterly distribution with information on seriousness. Data as available at time of database lock.

The available ADR data at the MPA are in two data formats—one historic with 90K reports, and a current with 19K reports (Fig 1). The formats differ and therefore, the data are used for different purposes. In the historic format, a report consists of a summary by the assessor, which includes the original report, but is not clearly formatted to reveal what was part of the incoming report, whereas the current data format contains the original free text fields including the description of adverse events with occasional notes from the assessors and the reported terms for the individual side effects. Hence, data in the historic format were only used for continued pre-training of the language model while reports in the current data format were used for the seriousness classification task.

For classification, to avoid bias in relation to medicinal product and patient characteristics, only the reported adverse event terms and free text description of the reaction are fed to the model. S1 Fig shows a histogram of the report length. All reports were anonymized through the removal of all digits from free text, effectively deleting any misplaced numbers directly or indirectly related to identity.

Due to the rapid increase in reported events related to the Covid-19 vaccinations from Q1 2021 onward, with strong class imbalances related to the priority in assessing events reported as serious, the data lock point for use in modelling was set to 2020-12-31.

The development dataset was split in a fixed training (80%) and validation (20%) dataset, used for training and validation of all models. After all models were frozen, all reports from Q1 and Q2 of 2021 (excluding those related to Covid-19 vaccines) were acquired for prospective testing. In addition, a test sample of 200 reports was reserved for benchmarking machine learning models against human assessors. Table 1 shows an overview of the datasets.

**Table 1:** Datasets used in the study.

Dataset	Data format	Usage	Time period	Serious	Non-serious	Total
Pre-training	Historic	Continued pre-training	2000-01-03–2017-12-29	35,781	48,208	89,712
Development	Current	Classification training and validation	2017-01-12–2020-12-31	5,557	9,428	14,985
Prospective	Current	Prospective testing	2021-01-01–2021-06-30	1,170	2,273	3,443
Human test	Current	Human benchmarking	2017-01-12–2021-11-09	93	107	200

### Models and training

**Fig 2:**
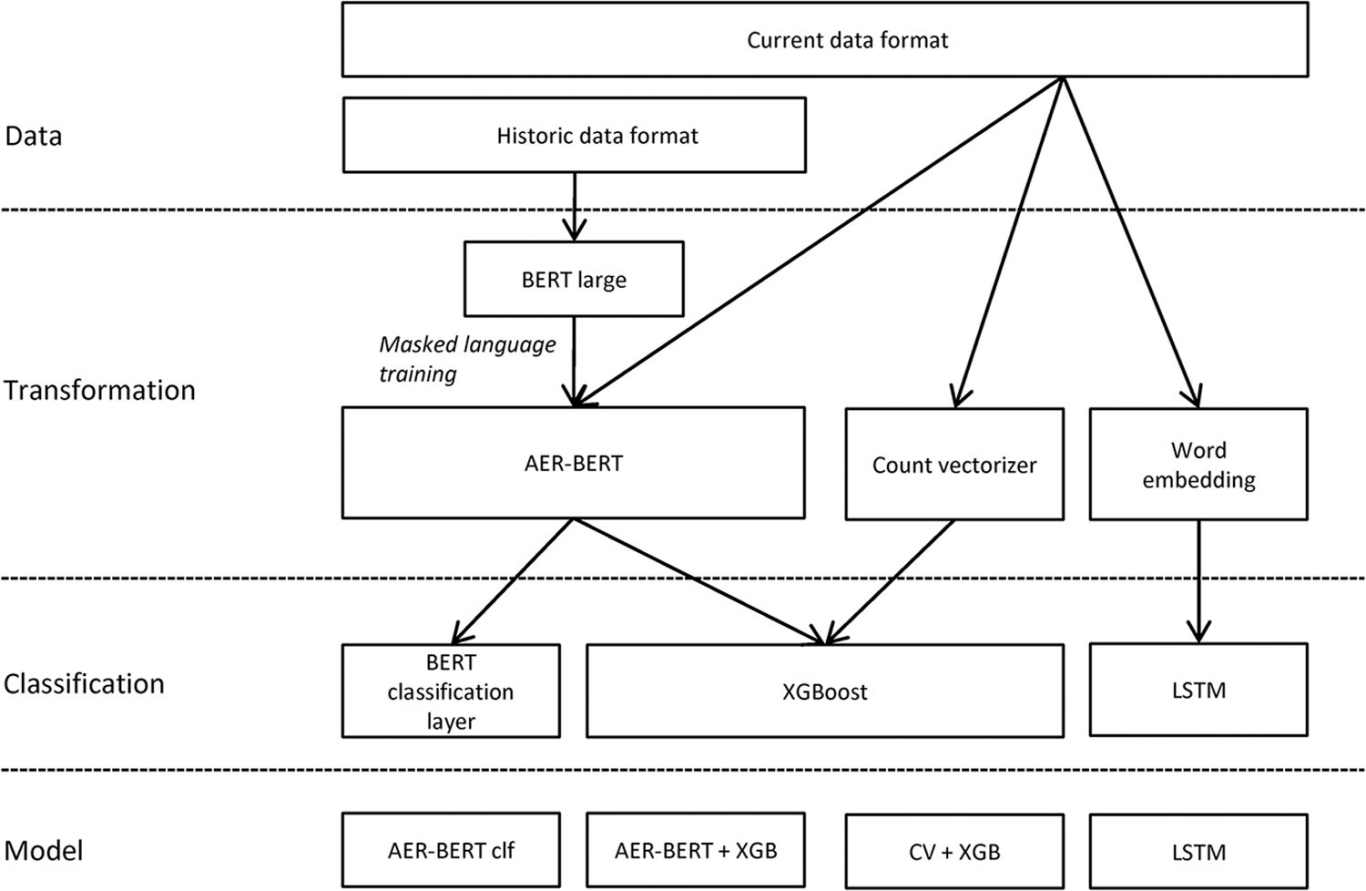
Overview of the data flow and model architectures investigated.

Four different classification models were investigated as described in Fig 2.

- Count vectorizer transformation with XGBoost classifier (CV+XGB)
- BERT embeddings with XGBoost classifier (AER-BERT+XGB)
- LSTM based deep neural network with word embeddings (LSTM)
- BERT for sequence classification (AER-BERT clf)

Python version 3.8.10 was used together with scikit-learn version 1.1.1, transformers version 4.17.0, sentence_transformers version 2.2.0, tensorflow version 2.9.1, tensorflow_text version 2.9.0, and pytorch version 1.11.0+cu113.

*XGBoost* (XGB) version 1.6.1 with *scikit-learn XGBClassifier API* was used to perform the classification task for CV+XGB and BERT+XGB. *XGBoost* is an optimized distributed gradient library with parallel tree boosting (i.e., a boosted random forest model).

The Swedish pre-trained BERT large model (340M parameters) from AI Nordics [7] was used for the BERT based models (AER-BERT+XGB and AER-BERT clf). BERT large models use 1024 dimensions to create the text embeddings. The *CrossEncoder* module from the *sentence-transformer* (ver. 2.2.0) library was used as data pipeline and trainer for AER-BERT classifier, feeding only single sequences during training.

#### Continued language model pre-training

The pre-trained BERT model from AI Nordics is trained on multiple Swedish data sources including FASS, an open resource containing product information (including ADR listings) for medicinal products authorized for use in Sweden. In addition to training the model for classification as is, it was investigated if a continued pre-training of the BERT language model on a domain specific text corpus (i.e., summaries of reports of suspected ADRs) could increase the performance. To this end, the language model was trained by masked language modelling on the historic report (pre-training) dataset.

All reports were concatenated and tokenized into sub-words. The tokenized data was split into 98,040 chunks of equal length (128 tokens) and 10% of the words were replaced by the mask token. The masking rate was set below the commonly used level of 15% [2,20] so as to not risk overfitting to the limited language training data set and losing general language capabilities from the original model. In line with Gu *et al*. [14] we chose to mask whole words rather than word pieces, as the reports contains many complex biomedical terms that the BERT tokenizer would split into a high number of sub-words with low semantic overlap with the original training language corpus.

The chunks were split into a training set (90%) and a validation set (10%). Perplexity (defined as the exponential of the cross-entropy loss) for the original model on the validation set was 3.01. The model was trained with an initial learning rate of 2e-5. Training was stopped at six epochs to not risk losing too much language knowledge from the initial model (see S2 Fig for perplexity curve). After six epochs the perplexity on the validation set had decreased to 1.19. The continued pre-trained language model, Adverse Event Report BERT (AER-BERT), was then used in training for the classification task for AER-BERT clf and AER-BERT+XGB.

#### Count vectorizer with XGBoost classifier (CV+XGB)

As a baseline, an XGB classifier was used with vector representations based on a bag-of-words approach. To transform the documents into vector representation the *scikit-learn CountVectorizer* was used, removing Swedish stop words and restricting the vocabulary to 10,000 features. The resulting word count vector representations were then used as feature input to fit the XGB classifier (see S1 Table for parameters).

#### BERT embeddings with XGBoost classifier (BERT+XGB)

For this model, sentence embeddings were used as input features for the XGB classifier.

*SentenceTransformer* from the *sentence_transformers* library was used to embed the reports. The *SentenceTransformer* loads the BERT model and generates sequence representations that are mean poolings of the token embeddings. The 1024-dimensional embeddings are then used as features to fit the XGB classifier (see S1 Table for parameters).

#### LSTM based deep neural network classifier (LSTM)

*Keras* (from *tensorflow* library) and *tf_text* (from *tensorflow_text* library) were used to implement the LSTM.

A vocabulary was constructed from the 20,000 most used words after removing Swedish stop words, together with 1,000 out-of-vocabulary buckets. From this, a static vocabulary table was created to encode words.

Using *Keras*, a sequential neural network model was set up, with an embedding layer of dimension 128, a bidirectional LSTM (Long Short-Term Memory) layer of size 128, a dense layer of size 128 with *ReLU* activation, and finally an activation layer of size 1 with sigmoid activation. The model was compiled with *Keras BinaryCrossentropy* loss function and *Keras* optimizer Adam with a learning rate of 5e-5. The model was trained for three epochs.

#### BERT for sequence classification (BERT clf)

The model was trained for 1 epoch. A post-hoc experiment confirmed that training for 2 epochs does not provide any notable increase in performance on the development set (see S3 Fig for loss curve). Ten percent of the training data was used for warm-up and the AdamW optimizer with initial learning rate of 2e-5 was applied.

## Results

### Development set

S4 Fig shows the distribution of predicted probabilities for class serious (1 = serious, 0 = non-serious) for the different models. It is clear from this figure that the BERT based models are more polarised in their predictions (also when they are incorrect), while the LSTM and most notably the CV+XGB model make predictions more evenly distributed and hence, fewer are completely wrong.

#### Monte Carlo cross-validation

**Fig 3:**
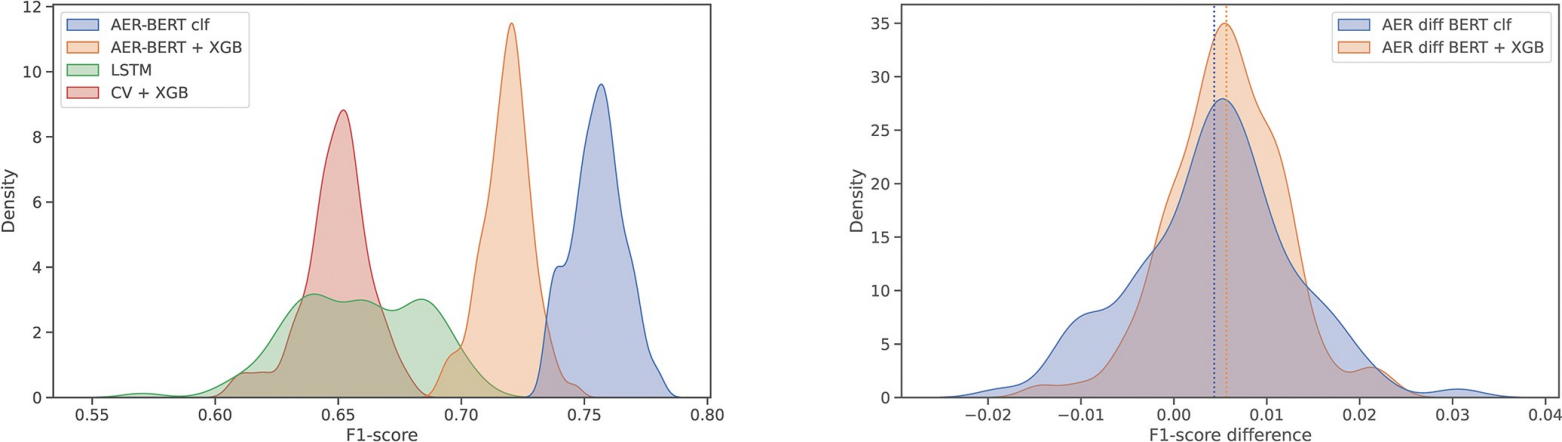
Density plots of F1 scores for all models (left) and F1 difference between models using BERT and AER-BERT (right).

To further compare the performance of the different models a bootstrapping technique was applied, where the development dataset was randomly split in 100 train and validation sets (80% / 20%). All models were trained and validated on each random set, allowing calculation of empirical confidence intervals (Table 2 and Fig 3).

**Table 2:** Average classification metrics comparison from Monte Carlo cross-validation.

Classifier	Accuracy Mean (SD)	Precision Mean (SD)	Recall Mean (SD)	Specificity Mean (SD)	F1 score Mean (SD)
AER-BERT clf	0.833 (0.0066)	0.828 (0.0216)	0.695 (0.0226)	0.915 (0.0141)	0.755 (0.0105)
BERT clf	0.831 (0.0060)	0.827 (0.0172)	0.688 (0.0224)	0.915 (0.0117)	0.751 (0.0110)
AER-BERT + XGB	0.811 (0.0066)	0.800 (0.0135)	0.652 (0.0128)	0.904 (0.0073)	0.719 (0.0099)
BERT + XGB	0.807 (0.0064)	0.795 (0.0133)	0.646 (0.0122)	0.902 (0.0072)	0.713 (0.0095)
LSTM	0.770 (0.0109)	0.737 (0.0445)	0.602 (0.0706)	0.869 (0.0473)	0.658 (0.0268)
CV + XGB	0.780 (0.0077)	0.793 (0.0158)	0.550 (0.0154)	0.916 (0.0065)	0.649 (0.0136)

To investigate if additional pre-training on domain specific language further increases performance, the AI Nordics (BERT) original model was compared with the further pre-trained language model (AER-BERT). Fig 3 (right panel) shows that using AER-BERT only marginally increases the F1 score and that the effect on the XGBoost based classifier is greater than on the BERT classifier.

### Prospective model testing

**Fig 4:**
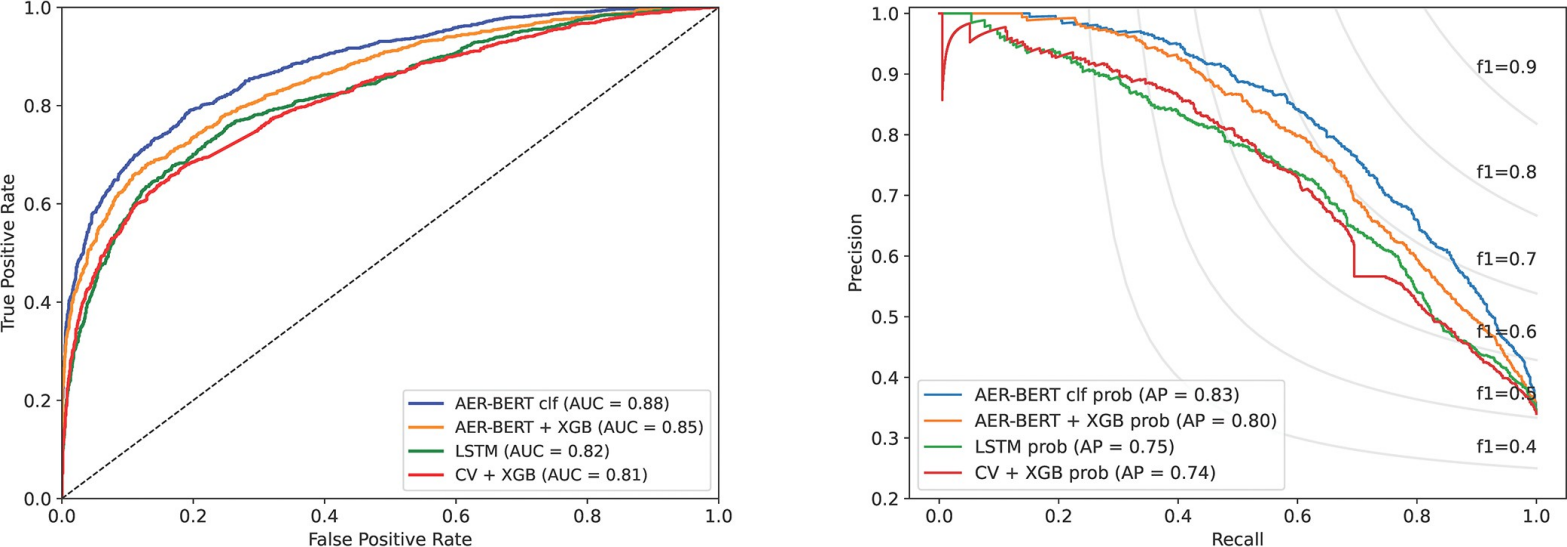
Receiver operating characteristics (left) and Precision-Recall curve (right) for the four model architectures on the prospective test set.

The final versions of each type were evaluated on the prospective test set, showing performance figures comparable to those seen during validation. The AER-BERT clf outperforms the other models on most classification metrics, with a F1 score of 72.1% (Table 3) and a ROC AUC of 0.88 (Fig 4, top left panel) and PR AUC of 0.83 (Fig 4, top right panel).

**Table 3:** Classifier metrics for the prospective test set.

Classifier	Accuracy	Precision	Recall	Specificity	F1 score
**AER-BERT clf**	0.827	0.796	0.658	0.913	0.721
**AER-BERT + XGB**	0.814	0.786	0.622	0.913	0.695
**LSTM**	0.789	0.743	0.578	0.897	0.650
**CV + XGB**	0.786	0.759	0.542	0.912	0.632

### Human performance benchmarking

To benchmark the models against human level performance, an external hold-out set of 200 reports was sent for blinded secondary assessment by two experienced human assessors. Assessors were presented with the same task as the prediction models–using adverse event terms and free text narrative from the original report to classify into serious and non-serious groups.

**Fig 5:**
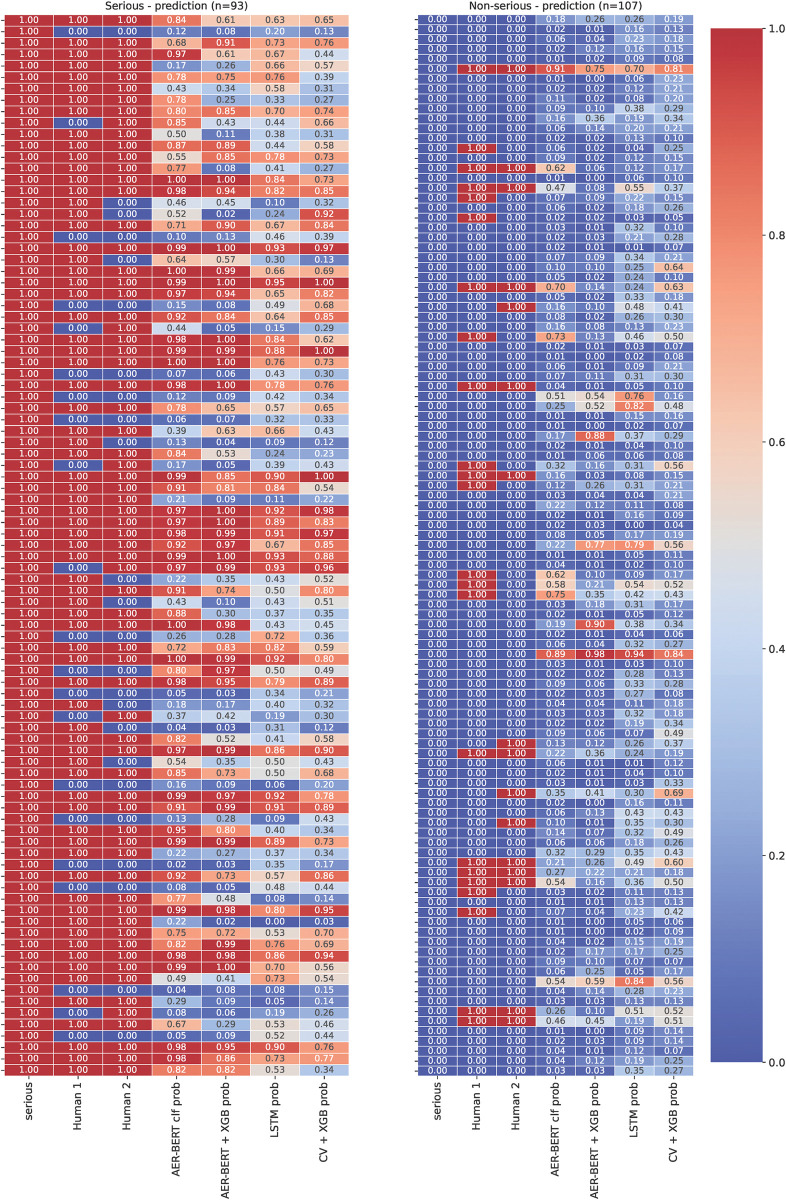
Results from hold-out sample with two human assessors and the four models. We show predictions on the serious (left) and non-serious reports (right) separately for each class, where the leftmost column in the prediction heatmap always corresponds to the database annotation.

Table 4 shows that the best model performs close to the human level. Fig 5 shows the individual predictions, including several instances where models and humans are in agreement with each other but not in line with annotation–this likely indicates annotation errors, as the regulatory practice during assessment is to never downgrade an event reported as serious although there is nothing in the reported terms or narrative that indicate that the criteria for a serious event is met.

**Table 4:** Comparison of model vs. human performance on the human test set (200 reports).

Classifier	Accuracy	Precision	Recall	Specificity	F1-score
**Human 2**	0.800	0.812	0.742	0.850	0.775
**Human 1**	0.780	0.758	0.774	0.785	0.766
**AER-BERT clf**	0.785	0.847	0.656	0.897	0.739
**AER-BERT + XGB**	0.760	0.869	0.570	0.925	0.688
**LSTM**	0.745	0.850	0.548	0.916	0.667
**CV + XGB**	0.720	0.785	0.548	0.869	0.646

## Discussion

In this study, we show that transformer-based language models can provide close to human-level performance to the task of triaging reports of suspected ADRs. Given the increasing number of reports globally and the need for preparedness for future surges in the number of reports–e.g., in relation to urgent public health interventions–we believe our results support implementation of AI in the field of regulatory pharmacovigilance, augmenting human assessors by providing automated detection of reports likely to be upgraded in seriousness during assessment.

Such reports can then be prioritised for earlier human assessment, allowing for faster signal detection in the post-authorisation phase of medicinal products. However, as the full process includes additional steps of assessment and annotation, and sometimes collection of additional data, there is still need for a human in the loop for each case to correct for model misclassifications and to provide ground truth annotation for downstream re-training of classification models.

The two BERT based models surpass both classical NLP techniques such as the bag-of-words approach and LSTM neural networks when applied to the current scale of training data, with the model using an internal classification layer slightly outperforming the XGBoost classification from BERT embeddings. The most likely reason why pre-trained models such as BERT outperform models without language pre-training, is that the BERT general language understanding capabilities with a self-attention mechanism provide a robustness that cannot be achieved when training simpler model architectures from scratch with our relatively small training data set. In summary, our findings are in line with previous learnings in the field [2].

The effect of continued language pre-training of the BERT model on performance is slightly more noticeable with the XGBoost classifier than with the BERT classifier. This is likely due to the fact that, when training the BERT classifier, the entire model is updated, which includes enhancing the domain language knowledge from the training dataset. In contrast, when training XGBoost classifier working on BERT embeddings, the BERT model itself is not updated and hence the BERT language pre-training on historic reports is more important.

One challenge posed by reports of suspected ADRs datasets is that an event reported as serious is considered regulatory true and is not downgraded even if an assessor deems it non-serious. In such cases, this additional information that the assessor disagrees with the original serious label was not captured in our current report processing workflow. A non-serious report, on the other hand, is upgraded if any of the seriousness criteria are met during assessment. This means that the dataset contains a proportion of mis-labelled reports that can confuse the models during training and impair an exact measure of model performance.

In summary, to further improve on the current approach, improvements in the input data workstream are needed. Increased data quality could potentially allow further increases in performance, and may be further improved through the use of larger language models such as GPT-3 that may capture the input language semantics in even higher detail although performance in the Nordic languages may not always surpass locally trained BERT models [6]. Furthermore, full-size GPT-3 models with 175B parameter require highly specialized hardware to run locally. Hence, they are currently most often accessed through a non-EU third-party provider, which limits the use to cases where data can be transferred freely.

Looking into the future, the field of NLP and machine learning holds promise for supporting additional steps of the process, such as named entity recognition for matching reported terms to the MedDRA standard and for performing automatic signal detection in databases of suspected ADRs.

## Supporting information

S1 Table XGBoost parameters. (PDF)

S1 Fig ADR report length histogram. (PDF)

S2 Fig Perplexity curve from the continued language model pre-training. (PDF)

S3 Fig Loss curve from BERT classification training. (PDF)

S4 Fig Distribution of predicted probabilities for class serious (1 = serious, 0 = non-serious) for the different models. (PDF)
