# Supplementary material for: BERT based natural language processing for triage of adverse drug reaction reports shows close to human-level performance
Source: PLOS Digit Health. 2023 Dec 6;2(12):e0000409. doi: 10.1371/journal.pdig.0000409 (PMC10699587; doi:10.1371/journal.pdig.0000409)
Supplement: S1 Table — (PDF) [file pdig.0000409.s001.pdf]

XGBoost parameters

Parameter	Value
**Count vectorizer**	
objective	multi:softprob
num_class	2
n_estimators	100 (default)
max_depth	6 (default)
learning_rate	0.3 (default)
subsample	1 (default)
colsample_bytree	1 (default)
reg_alpha	0 (default)
reg_lambda	1 (default)
**BERT embeddings**	
objective	multi:softprob
num_class	2
n_estimators	200
max_depth	12
learning_rate	0.2
subsample	0.8
colsample_bytree	0.8
reg_alpha	8
reg_lambda	2
