# Supplementary figures and images for: BERT based natural language processing for triage of adverse drug reaction reports shows close to human-level performance
Source: PLOS Digit Health. 2023 Dec 6;2(12):e0000409. doi: 10.1371/journal.pdig.0000409 (PMC10699587; doi:10.1371/journal.pdig.0000409)

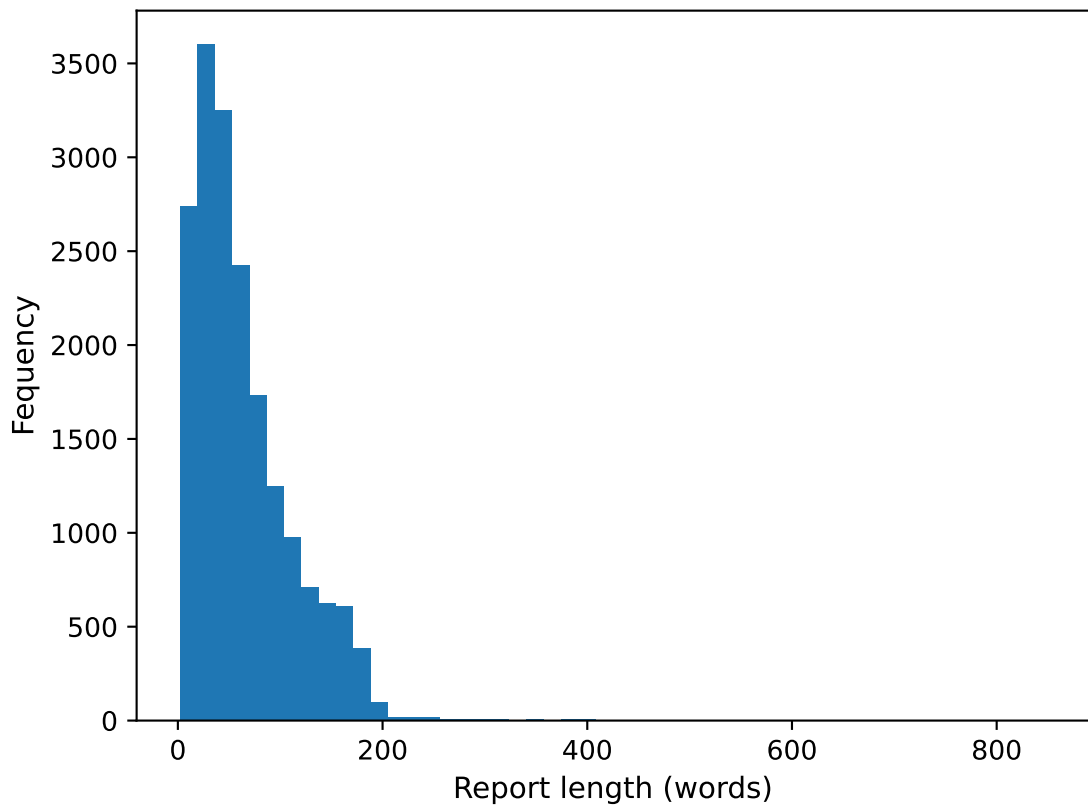

Supplement: S1 Fig — (PDF) [file pdig.0000409.s002.pdf]

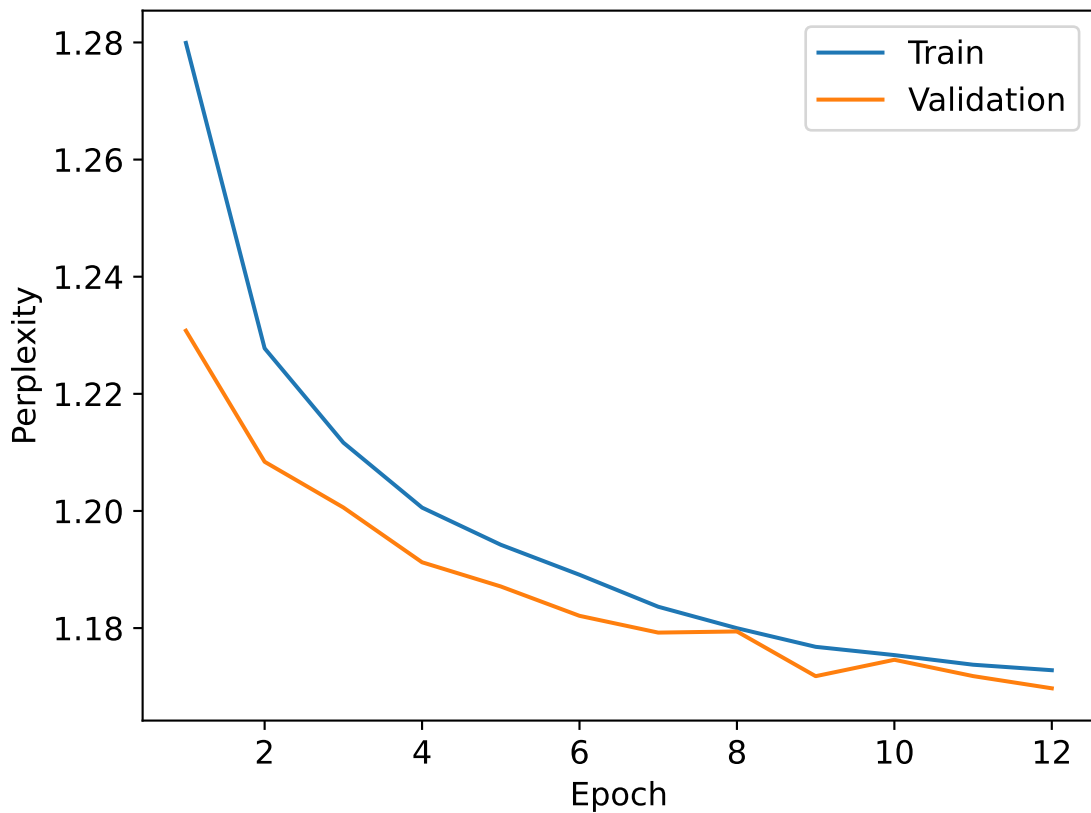

Supplement: S2 Fig — (PDF) [file pdig.0000409.s003.pdf]

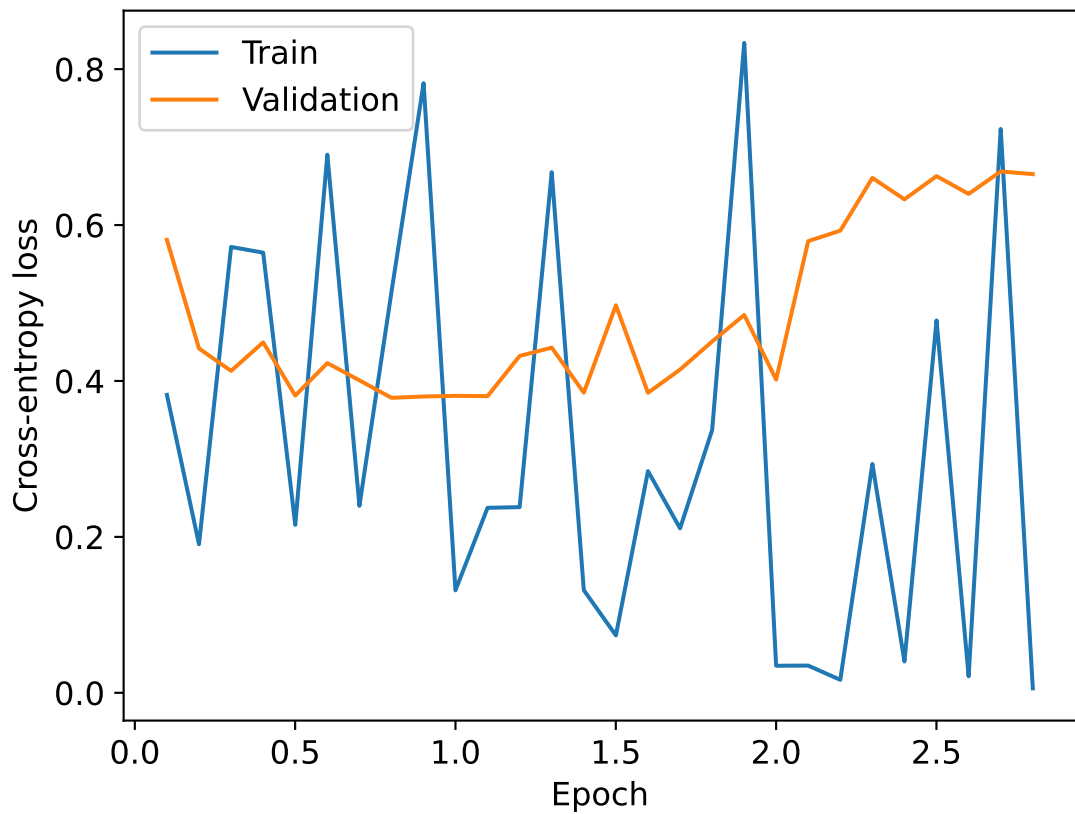

Supplement: S3 Fig — (PDF) [file pdig.0000409.s004.pdf]

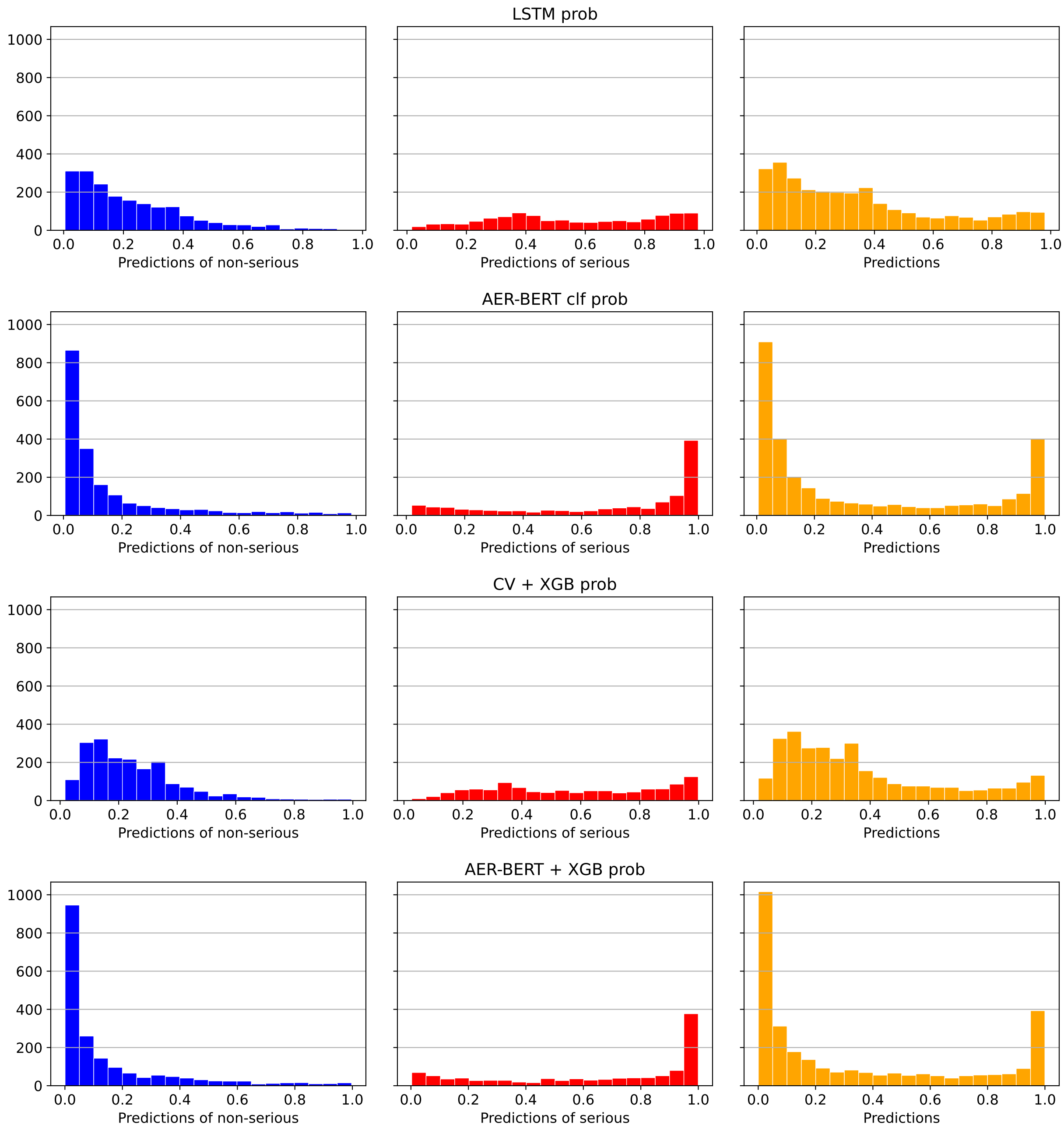

Supplement: S4 Fig — (PDF) [file pdig.0000409.s005.pdf]
